# *CAD* gene and early infantile epileptic encephalopathy-50; three Iranian deceased patients and a novel mutation: case report

**DOI:** 10.1186/s12887-022-03195-4

**Published:** 2022-03-11

**Authors:** Sepideh Gholami Yarahmadi, Saeid Morovvati

**Affiliations:** School of Advanced Sciences and Technology, Islamic Azad University-Tehran Medical Sciences, Tehran, Iran

**Keywords:** CAD, EIEE50, Epilepsy, Seizure, Encephalopathy

## Abstract

**Background:**

Early infantile epileptic encephalopathy is a severe form of epilepsy that is genetically extremely heterogeneous and characterized by seizures or spasms at the beginning of infancy. Homozygous or compound heterozygous mutation in the *CAD* gene cause early infantile epileptic encephalopathy-50 (EIEE50). This case report describes the clinical and molecular features of three patients affected with early infantile epileptic encephalopathy.

**Case presentation:**

In this report, we describe the clinical features of two deceased daughters and one recently deceased son affected with seizure, muscular hypotonia, and developmental delay. After genetic counseling, blood samples were obtained from the parents, and whole-exome sequencing was performed. Genomic DNA was extracted from whole blood, and mutation analysis was performed using PCR and sequencing methods for the CAD gene. Genetic analysis using the whole-exome sequencing method has detected a novel likely pathogenic mutation on *CAD* gene, c.2995G > A (p.Val999Met), in heterozygous states in asymptomatic parents and homozygous state in affected newborn son. This mutation has not been reported in the literature for its pathogenicity.

**Conclusions:**

The asymptomatic parents are carriers for the likely pathogenic variant in the CAD gene, and the recently deceased newborn son had the same mutation in a homozygous state. Given that, multiple lines of in silico computational analysis support the detrimental impact of the variant on the gene, and this variant is absent in population databases. Pathogenic mutations in the CAD gene are related to autosomal recessive EIEE50 with similar signs and symptoms to our patients. Ultimately, it is confirmed that this mutation is causative in our patients.

## Background

One the most drastic form of epilepsy, which is extremely heterogeneous to the extent that it consists 67 types engendered by a mutation in diverse genes is Early Infantile Epileptic Encephalopathy. One of the main causes of Early Infantile Epileptic Encephalopathy-50 (EIEE50) (#616457) is homozygous or compound heterozygous mutation in the CAD gene (#114010). There are different signs and symptoms indicating EIEE50 appearing in the first year of life, at the beginning of infancy is being indicated by spasms or frequent tonic seizures [1]. In addition, EIEE50 is an autosomal recessive disorder determined by severe developmental regression, delayed psychomotor development, early-onset seizures, and normocytic anemia. CAD is a single large polypeptide with three highly conserved enzymatic activities: carbamoyl phosphate synthase (CPS2), aspartate transcarbamylase (ATCase), Dihydroorotase (DHOase); these are the first three enzymes of de novo pyrimidine, and the name of CAD is the abbreviation of these enzymes [2]. Human genetic disorders rarely occur in the last three steps of the de novo pathway. The de novo synthesis of pyrimidine nucleotides is essential for the proliferation of mammalian cells [3, 4]. The pyrimidines are mainly utilized for de novo DNA and RNA synthesis [5, 6]. CAD encodes a multi-functional enzyme complex that catalyzes the first stages of biosynthesis of de novo pyrimidine, and mutation in the CAD gene can impair de novo pyrimidine biosynthesis between these genes. Approximately 2% of the human genome is estimated to encode glycosylation-related proteins, and mutations in over 100 genes produce congenital disorders of glycosylation [7]. All the cells in the body require de novo synthesis of RNA and DNA, therefore, CAD expression must be ever-presented since The CAD gene is located at 2p23.3 and consists of 7131 bp and 45 exons [8]. More than 250 pathogenic and likely pathogenic mutations have been reported in the CAD gene up to now. This case report presents two affected deceased daughters and one newborn affected son with a novel mutation in the CAD gene.

## Case presentation

**Fig. 1:**
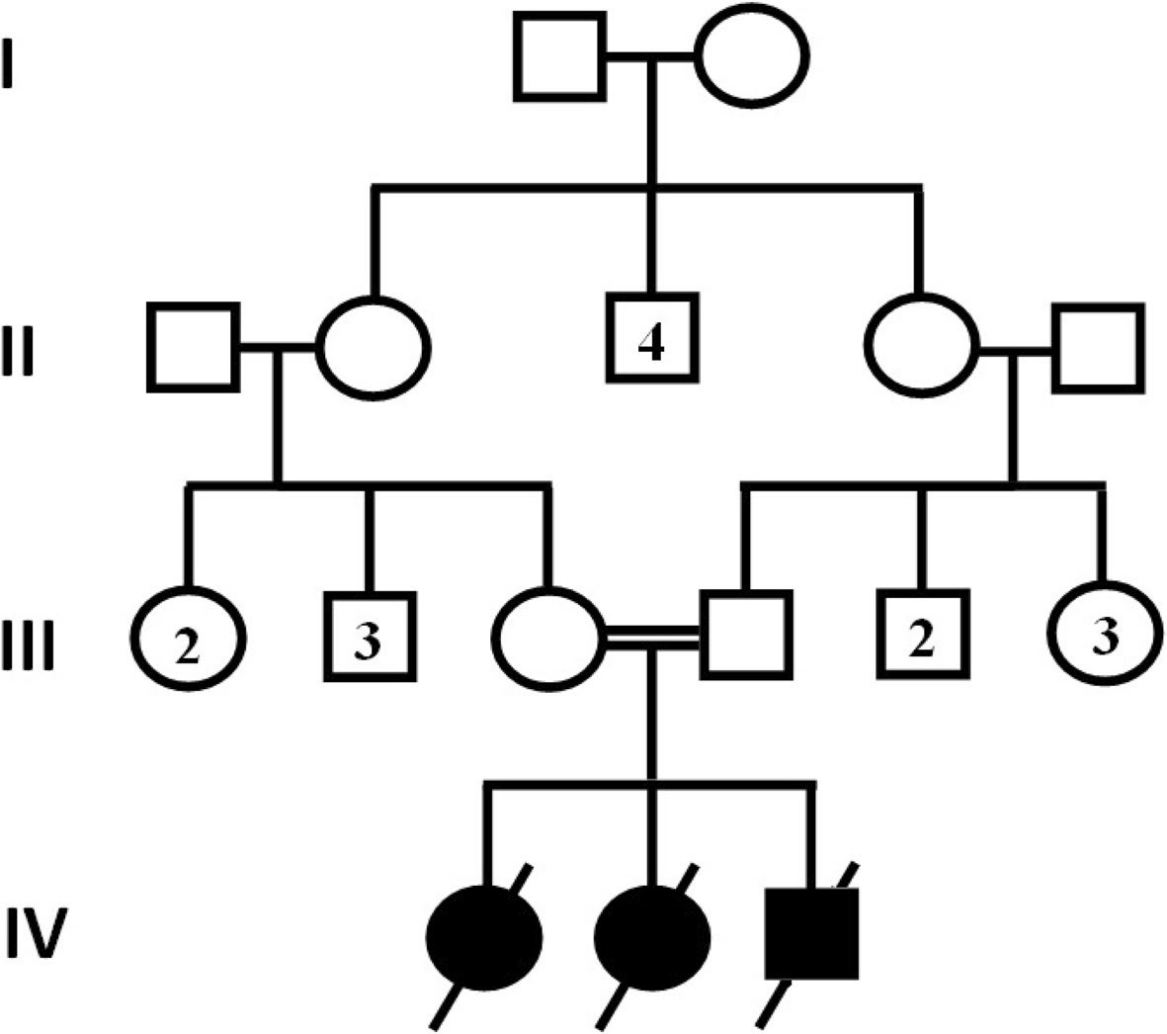
Family pedigree

A young first cousins couple with two affected deceased daughters were referred to our center. The father of the family was 44 years old and the mother of the family was 36 years old. Initially, the couple was provided with genetic counseling, and as shown in Fig. 1, their family pedigree was drawn.

Informed consent for genetic studies of participants was obtained, and the procedure was followed by the guidelines of Ethics Committees. Since there was no access to deceased patients, genetic analysis was performed on the mother using whole-exome sequencing, then the variants found in the mother were investigated in the father. Subsequently, with the birth of another affected child, the likely pathogenic mutation was found in the mother, and it was confirmed in her spouse and examined in the affected newborn.

In this report, we describe the clinical feature of the patients, molecular analysis of their parents, and the affected newborn son. In all of the patients, pregnancy and delivery were unremarkable. They were born with normal birth weight, and length and both of them showed regular facial and physical features at birth.

### Case 1

A female was the first child born to consanguineous healthy Iranian parents. After 7 days, the seizure occurred along with head dropping, jerks and twitches of hands, body stiffening, turning eyes to one side, and epileptic spasms. The seizure lasted less than 1 minute. At first, the seizure began as she had woken up and cried. But after a few days, she was not reacting and responding like a normal infant. At 25 days, antiepileptic medication such as Phenobarbital was started, and seizures were controlled entirely until the age of 10 months. But after that, despite multiple antiepileptic drug treatments, seizures began again. She underwent PDA surgery to close the blood vessel at age 3 months due to suffering from PDA and cyanosis. She was unable to breastfeed. Also, she did not have head control. Muscular hypotonia was one of the most important signs in the patient, impeding her to sit and walk independently to the extent by which she was even unable to crawl until death. In addition, she had a developmental delay. Her speech was very feeble, with little vocalizations. She did not start teething until she was 1 year old. At the age of 8 months, after occupational and language therapy, she could laugh, cry and swallow. Seemingly, she had been recovering gradually. But after a while, the seizure occurred again, and she lost her acquired skills altogether. At the age of 2 years, the duration of seizures increased. She had mental problems, and her cognitive skills were weak. At this age, she had difficulty in eating and swallowing, and she was constantly coughing. Also, the size of her body parts were abortive and too small, and generally her physical development such as body weight and height had been hindering significantly in comparison to the normal standards of her age at the time of death. In the last days of life, strabismus was observed, and all of the signs and symptoms intensified, and ultimately she died at the age of 3 years and 8 months.

### Case 2

A female, who was the younger sister of case number 1. The first seizure occurred at 3 days. Like her sister, she was suffering from hypotonia and could not breastfeed, cry, laugh, walk, sit, and crawl. She had had a poor interaction with others. Also, she had not been responding to painful stimuli. She did not teethe. The signs and symptoms were more severe than her sister’s, and finally, she died at the age of 1 year and 13 days.

### Case 3

A male was the third child of the family. He was born when his two sisters had died already, and the molecular analysis had been performed on his parents. At 2 days, the seizure occurred like his sisters and antiepileptic medication like phenobabital was started. During the seizure, the muscle tone was significantly increased in the body, arms, or legs, causing sudden stiffening movements. The breathing decreased or ceased altogether, causing cyanosis of the lips and face, turning eyes to one side, and epileptic spasms. The seizure lasted about 1 minute. At first, he had a seizure 6 times a day. He had severe seizures at the age of 7 months that caused him to forget all the acquired skills. After that, antiepileptic medication such as phenytoin and levetiracetam was started, and as a consequence, the seizures were partially controlled. He cannot breastfeed and has the same problems like controlling his head, ordinary behavior such as laughing and crying like his sisters, and he does not start teething until 1 year and 3 months. He had developmental delay as well. Also, in brain MRI there was a generalized widening of extra-axial spaces. In general, although drug dose changes was controlling seizures, all of the signs and symptoms had intensified in his case until death compared to his former sisters. Having the exact same sypmtoms and signs like his former sisters, he recently died at the age of 2 years and 6 months.

Because the affected daughters had died and there was no access to their DNA sample, the whole-exome sequencing (WES) was performed on the mother of the cases. DNA extraction from whole blood. Library preparation was performed using Twist Core Exome kit (TWIST Bioscience, USA, https://www.twistbioscience.com/) through the manufacturer’s instruction. Sequencing of libraries was performed by high-throughput paired-end sequencing using NovaSeq sequencing platform (Illumina Inc., CA, USA). Interpretation of detected variants in terms of pathogenicity was done based on American College of Medical Genetics and Genomics (ACMG) guideline. The identified variant was evaluated for disease-causing nature using the Mutation Taster (http://www.mutationtaster.org) and UniProt database (www.uniprot.org). Sanger sequencing method was used to confirm the identified mutations in the mother and father of the cases, and subsequently in affected newborn son.

**Fig. 2:**
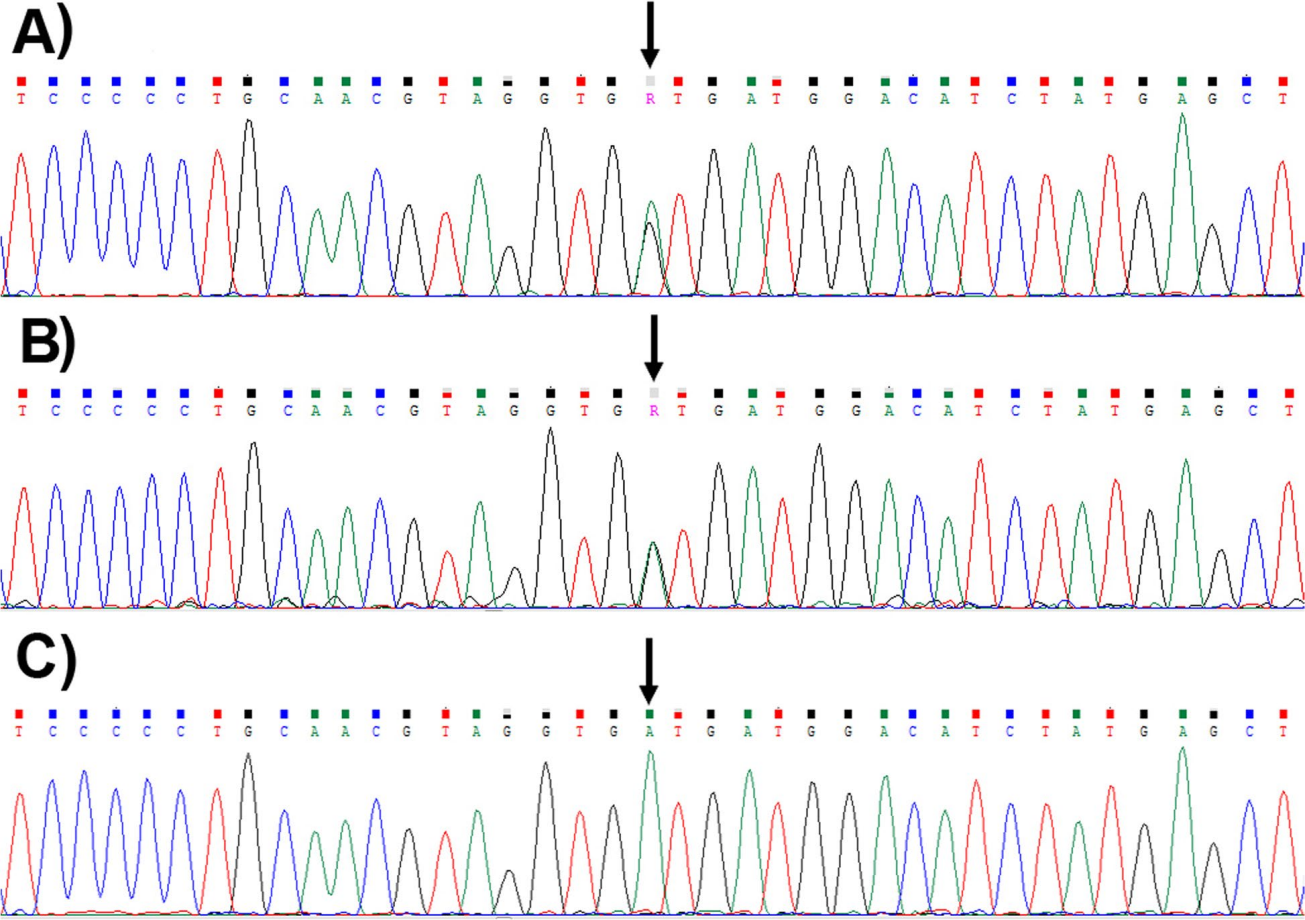
Chromatogram in the father (**A**) and mother (**B**) of the patients are showing the c.2995G > A (p.V999M) mutation in CAD gene in heterozygous states. The chromatogram in the affected child (**C**) shows the mutation in a homozygous state

Exome sequencing in the mother identifies five heterozygous likely pathogenic mutations in the following genes: *ADD3*, *AP3B2*, *ARHGEF2*, *DNM2,* and *CAD*. Sanger sequencing method in the father for the mentioned variants detected only the reported variant in the *CAD* gene. This heterozygous likely pathogenic mutation at exon 20 of *CAD* gene (c.2995G > A or p.Val999Met) is associated with early infantile epileptic encephalopathy-50 see Fig. 2. This mutation has not been reported in the literatures for its pathogenicity; however, multiple lines of in silico computational analysis support the deleterious effect of the variant on the gene or gene product(s). This variant is absent in population databases. Consequently, investigation of this variant in the affected newborn has shown it in a homozygous state as shown in Fig. 2.

## Discussion and conclusions

A homozygous missense mutation in CAD gene, c.98 T > G (p.Met33Arg), was indicated in four children with clinical manifestation of the neurometabolic disorder including epileptic encephalopathy, global developmental delay, epileptic encephalopathy, anemia and seizures with anisopoikilocytosis, in a research conducted by Johannes Koch and his colleagues [9].

Ng et al. characterized an individual who was compound heterozygous for mutations in different domains of the *CAD* gene. One mutation, c.1843-1G > A, resulted in an in-frame deletion of exon 13, and the other, c.6071G > A, caused a missense mutation (p.Arg2024Gln) in a highly conserved residue that is essential for the carbamoyl-phosphate binding that is required for de novo pyrimidine biosynthesis. Their patient showed delays in expressive language, mild hypotonia, and a slightly wide-based gait, developmental delay, and seizures noted at 17 months of age [10].

Recently, Russo et al. identified a case of syndromic congenital dyserythropoietic anemia due to the homozygous mutation p.Arg1789Gln in CAD gene, a 6.5-year-old boy with mild macrocytic anemia and suffering from epilepsy [11].

In this report, we described three patients with early infantile epileptic encephalopathy-50 caused by novel *CAD* mutation. Comparing our cases with patients reported by Koch et al. with another mutation in the *CAD* gene indicates that several main phenotypic features such as intractable seizures, hypotonia, loss of acquired skills, minimally conscious state, and intellectual disability are the same in all patients. Unlike our patients, in which signs of the disease appeared from the beginning of infancy, it appeared later in the patient reported by Ng et al..

Since pathogenic mutations in the CAD gene are related to autosomal recessive early infantile epileptic encephalopathy-50 (EIEE50) with similar signs and symptoms to those of our patients. In has to be mentioned that both daughters had been deceased before their parents were examined by doctor for the possible genetic disease. In addition, all the clinical signs and symptoms of the recently deceased son who were as same as the deceased daughters completely. Furthermore, the son was examined for all the involved genes in EIEE50 disease like his parents, and no mutation was detected in any gene except the CAD gene. Therefore, based on mentioned evidences, it is confirmed that the CAD gene mutation had caused the same disease in all three cases. Ultimately, this mutation can be used with caution in Prenatal Diagnosis (PND) and Preimplantation Genetic Diagnosis (PGD), and prevention of the birth of an infected child in their future pregnancy in this family and all the same cases worldwide.

## Data Availability

The datasets used during the current study are available in NCBI/Clinvar website through [PRJNA810706], [SAMN26274384] or [SRR18192541] accession numbers.

## Abbreviations

EIEE50: Early Infantile Epileptic Encephalopathy-50
CAD: Carbamoylphosphatesynthase, Aspartate transcarbamylase, Dihydroorotase
CPS2: Carbamoyl Phosphate Synthase
ATCase: Aspartate Transcarbamylase
DHOase: Dihydroorotase
PDA: Patent ductus arteriosus
WES: Whole-exome sequencing
ACMG: American College of Medical Genetics and Genomics
